# Duration and determinants of birth interval among women of child bearing age in Southern Ethiopia

**DOI:** 10.1186/1471-2393-11-38

**Published:** 2011-05-20

**Authors:** Samuel Yohannes, Mekitie Wondafrash, Mulumebet Abera, Eshetu Girma

**Affiliations:** 1 Hossana College of Health Sciences, Hossana, Ethiopia; 2 Department of Population and Family Health, Jimma University, Jimma, Ethiopia; 3 Department of Health Education and Behavioral Sciences, Jimma University, Jimma, Ethiopia

## Abstract

**Background:**

Longer intervals between consecutive births decrease the number of children a woman can have. This results in beneficial effects on population size and on the health status of mothers and children. Therefore, understanding the practice of birth interval and its determinants is helpful to design evidence based strategies for interventions. The objective of this study was to determine duration and determinants of birth interval among women of child bearing age in Lemo district, southern Ethiopia in March 2010.

**Methods:**

A community based cross sectional study design with stratified multistage sampling technique was employed. A sample of 844 women of child bearing age were selected by using simple random sampling technique after complete census was conducted in selected kebeles prior to data collection. Structured interviewer administered questionnaire was used for data collection. Actual birth interval was measured with the respondents' memory since majority of the women or their children in the area had no birth certificate.

**Results:**

Majority (57%) of women were practicing short birth interval length with the median birth interval length of 33 months. Actual birth interval length is significantly shorter than preferred birth interval length. Birth interval showed significant variation by contraceptive use, residence, wealth index, breast feeding and occupation of husbands.

**Conclusion:**

low proportion of optimal birth spacing practices with short actual birth interval length and longer preferred birth interval lengths were evident among the study subjects. Hence interventions to enhance contraceptive utilization behaviors among women in Lemo district would be helpful to narrow the gap between optimal and actual birth spacing.

## Background

Birth interval is the length of time between two successive live births [1]. Beginning with a live birth, the birth interval can be divided into the period of postpartum amenorrhea, the menstruating interval, and the following period of gestation [2]. Ethiopia is the second most populous country in Africa next to Nigeria, with population size of 73,918,505 million and total fertility rate of 5.4 [1,3]. Like many other African countries, Ethiopia has so far shown little change in fertility [5,6].

Fertility is an important component of population dynamics which plays a major role in changing the size and structure of a given population [7]. Factors affecting fertility are broadly classified into proximate (direct) and distal (indirect) factors. The proximal factors are bio-behavioral factors, like being sexually active, use of contraceptive, duration of postpartum infecundability, abortion and sterility which affect fertility directly, whereas, distal determinants, are socio-cultural factors which affect fertility indirectly through affecting the bio-behavioral factors [4,8,9].

Optimal spacing until the next pregnancy is generally understood to refer to resting period between pregnancies that allows the mother time to recover from pregnancy, labor and lactation [10]. Longer time period between births allows the next pregnancy and birth to occur more likely to be at full gestation and growth [11,12]. For years, family planning programs have advocated two years intervals between births for infant and child health and survival [11,12]. Recent research found that birth intervals of 3 to 5 years are safer for mothers and babies compared to birth intervals of two years or less [7,8,12]. Some researchers also opined that birth intervals longer than five years are less healthy suggesting that such mothers may lose the protective benefit of previous child bearing and hence have complications as seen in primigravida [12]. Few people including women themselves understand the risks involved in bearing children [7]

Women in developing countries have shorter birth intervals than they would prefer. The main reason for short birth intervals is that many women in developing countries do not use contraception after birth and therefore are likely to become pregnant once fecundity returns [13]. Birth spacing is a well-known, underutilized, and admittedly not fully understood health intervention [14]. Adequate child spacing is considered as a positive factor on the health of mothers and their children. A variety of demographic and socio economic characteristics influence women's spacing practices [7,15]. Thus, understanding practice of birth interval and factors which influence women's birth interval is critical for countries like Ethiopia with a population policy aiming at reducing fertility. Hence, this study was conducted to determine duration and determinants of birth interval among women of child bearing age in Lemo district, southern Ethiopia in March 2010.

## Methods

A community based cross sectional study was conducted in March 2010 in Lemo district. The study area is found in southern Ethiopia. It is located 230km south of the capital (Addis Ababa). According to 2005 census, female population in Lemo Woreda was 215,265. A sample of 844 women of child bearing age who experienced at least two deliveries and at least the last delivery being within the last five years prior to the data collection were included in the study. The sample size was determined with single population proportion formula by considering 50% proportion of women 15 - 49 years who spaced consecutive births 3 to 5 years (to obtain maximum sample size) with 95% confidence interval and design effect of 2. The study subjects were selected by using simple random sampling technique after a census was conducted in selected kebeles (the lowest administrative unit). The kebeles were stratified as urban and rural kebeles. Probability to proportional size allocation technique was used in the determination of the number of kebeles and study units included in each stratum. Two urban and six rural kebeles were randomly selected by using lottery method out of 8 urban and 35 rural kebeles. Because of 33 respondents were either absent or refused to participate in the study the response rate of the study was 96%.

In this study we measured birth history and interval using 8 items, socio-demographic 26 items and behavioral variables 19 items and the internal reliability (coefficient alpha) of the instrument was 0.73. The knowledge level of the respondents was assessed for the advantages of practicing optimal birth spacing and the disadvantages of practicing short birth intervals both for the mother and the child using 7 items. Those respondents who scored at least 60% of the knowledge questions correctly and the right optimum number of months or years between two successive births were considered as knowledgeable. Actual birth interval was measures with the respondents' memory since majority of the women or their children in the area had no birth certificate. Since actual birth interval was measured with the respondents' memory we studied women with history of at least two deliveries and at least one of the births within the last five years to minimize the recall bias. We only measured the interval between the last child and the preceding child. Birth interval among the respondents has been tri-chotomized based on recent recommendations into short birth interval (less than 36 months), optimum birth interval (36 to 60 months) and long birth interval (above 60 months) categories [11,16].

Data was collected by trained female data collectors using interviewer administered questionnaire adapted from different literatures (see additional file 1). The questionnaire were translated first to Hadiyigna (the local language) and back translated to English language to check conceptual equivalence. Supportive supervision was carried out during the entire data collection period.

Data was checked for completeness and analyzed using SPSS for windows ver. 16.0 (SPSS, Inc, 2007). First descriptive statistics was used to present the frequencies, of variables and followed by bivariate and multivariate analysis in order to see statistical association between the outcome and explanatory variables. Variables which showed significant association in the bivariate analysis were entered into multivariate logistic regression. Ethical approval was obtained from the Ethical Committee of Jimma University. Informed consent was obtained from the participants.

## Results

### Socio-demographic characteristics

out 811 study population, 552 (68.1%) were rural and the rest 259 (31.9%) were urban residents. The majority of the respondents 499(61.20%) were in the age ranges of 25-34 years. The mean and median age of the respondents was 31 (SD = 10.6) and 28 years respectively (Table 1)

**Table 1:** Socio-demographic characteristics of the respondents in Lemo district, Ethiopia, 2010

Variable	Categories	Frequency(n = 811)	%
**Residence**	Rural	552	68.1
	Urban	259	31.9

**Marital status**	Married	777	95.8
	Others*	34	4.2

**Religion**	Protestant	563	69.4
	Orthodox	121	14.9
	Islam	56	6.9
	Others	71	8.8

**Ethnicity**	Hadiya	678	83.6
	Amhara	44	5.4
	Kembata	42	5.2
	Others**	47	5.8

**Maternal education**	Cannot read and write	207	25.5
	Primary	444	54.8
	Secondary & above	160	19.7

**Husband's education**	Cannot read and write	82	10.1
	Primary	356	43.9
	Secondary & above	339	41.8

**Maternal occupation**	House wife	727	89.6
	Employee	24	3.0
	Others***	60	7.5

**Maternal age**	15-19	4	0.5
	20-24	47	5.8
	25-29	273	33.7
	30-34	223	27.5
	35-39	188	23.2
	40-44	38	4.7
	45-49	38	4.7

*divorced, widowed **Wolayta, Gurage, Dawro ***merchant, pension, farmer

### Birth spacing knowledge

Among the total respondents, 762 (94%) have ever heard about optimum birth interval between live births. Among those who had the awareness, two hundred forty (31.5%) reported the optimum birth interval between two successive births to be below 36 months and 454(59.6%) of them reported it to be between 36 and 60 months. The rest are in the category greater than 60 months. Sixty percent of the study participants were Knowledgeable about optimum birth spacing. The results showed that almost all, 806 (99.4%) of the respondents, reported the presence of health advantages of practicing optimal birth interval and 807 (99.5%) reported the presence of negative consequences of practicing short birth interval.

### Practice on birth spacing

**Figure 1:**
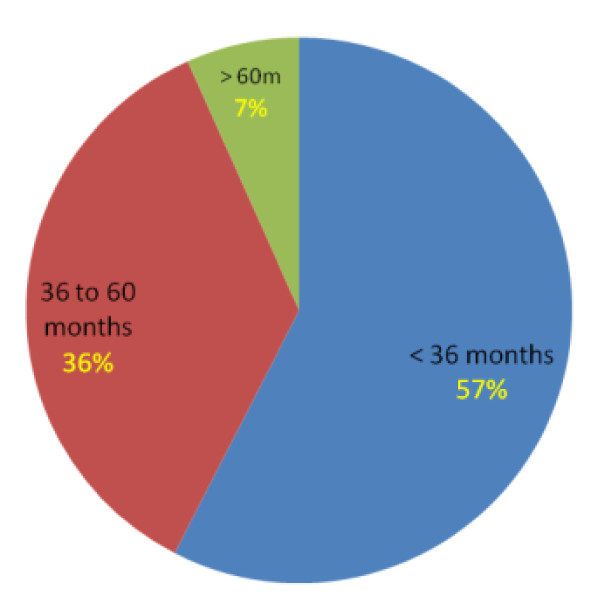
Proportions of mothers with their respective actual birth interval practice in Lemo district, Ethiopia, 2010

More than half 467(57.6%) of the study subjects had short birth interval. Two hundred ninety (35.8%) respondents had optimum birth interval and the remaining had long birth interval (figure 1). The median duration of actual birth interval was 33 months (SD+/-16.7) whereas; the median duration of preferred birth interval was 38 months (SD+/_19.1) for the last two successive births. The semi interquartile range is 4.5 months.

### Difference between actual and preferred birth interval

The difference between the means of actual and preferred birth interval length is found to be statistically significant (t = 14.2, df = 810, p-value <0.001, 95%CI (8.4, 6.1). This shows that the mean preferred birth interval length is significantly greater than the mean actual birth interval length.

### Intention to become pregnant

The desire to have more children before the conception of the last child was asked. Accordingly, 632 (77.9%) of the respondents had the desire to have the last child. Of those who wanted more children, 448 (70.9%) wanted to become pregnant then and 184 (29.1%) desired to postpone the pregnancy to sometime later. Among others, problems associated with contraceptive usage 140 (17.3%) and not using modern family planning methods 39 (4.8%) were the main reasons for failing to postpone the last pregnancy. Among 427 family planning users, 132 (30.9%) had become pregnant prior to the time they desired between the last two pregnancies. Forty eight (11.2%) of the users became pregnant while wanting to limit child bearing.

### Predictors of the birth interval length

Rural women were more likely to have short birth interval than urban women [OR = 2.7 (95%CI: (1.4, 5.1)]. Compared to being employee, daily laborers and students had high chance of having short birth interval. Similarly as the duration of breast feeding increases, the probability of short birth interval also increases. Women who were not using contraceptives are [OR = 1.6(95%CI: 1.1, 2.2)] times more likely to give birth within short period of time than users. Women with highest wealth quartile were 51% more likely to have short birth interval than with the lowest wealth quartile (Table 2)

**Table 2:** Predictors of birth interval in Lemo district, Ethiopia, 2010

Variable	Short birth interval		AOR* (95.0% C.I)
		
	No	Yes	
**Residence**			
Urban	135 (52.1)	124(47.9)	1
Rural	209(37.9)	343(62.1)	2.66(1.39, 5.08)
**Husbands' occupation**			
Employee	76(51.7)	71(48.3)	1
Farmer	167(38.8)	263(61.2)	0.81(0.38, 1.72)
Merchant	48(41.4)	68(58.6)	1.12(0.60, 2.06)
Daily laborer	26(37.7)	43(62.3)	0.13(0.03, 0.61)
Student	27(55.1)	22(44.9)	0.50 (0.26, 0.99)
**Duration of breast feeding**			
0 - 6 months	5(29.4)	12(70.6)	1
7 - 12 months	5(5.2)	91(94.8)	8.78(1.73, 44.66)
13 - 23 months	11(7.0)	147(93.0)	4.56(1.11, 18.69)
24 and above	323(59.8)	217(40.2)	0.25(0.07, 0.89)
**Contraceptive use before the conception of the last child**			
Yes	217(48.5)	230(51.5)	1
No	127(34.9)	237(65.1)	1.56(1.10, 2.21)
**Wealth index**			
Lowest quartile	65(30.4)	149(69.6)	1
Second quartile	73(41.2)	104(58.8)	0.75(0.45, 1.26)
Third quartile	92(42.4)	125(57.6)	0.68(0.42, 1.12)
Highest quartile	114(56.2)	89(43.8)	0.49(0.25, 0.96)

* *Adjusted for some selected socio-demographic variables*

## Discussion

Since the area is remote to the central part of the country, 60% knowledgeable mothers about birth spacing is a better finding. Population report of 2002 in 55 sub-Saharan Africa showed that 57% of women of child bearing age practiced birth interval lengths less than 3 years [16]. A finding of this study is similar with this situation in which case 57% of the respondents had less than 3 years. On the other hand, only 36% of the respondents were currently practicing optimal birth interval length. This is far lower relative to higher proportion (60%) of the respondents who were considered knowledgeable. This could be due to the fact that about 31% of contraceptive users became pregnant before the time they desired it and this could have greatly contributed to the high proportion of mothers with short birth interval. The median length of actual birth interval is also in line with the findings of most of the studies done before in other places [1,16,17].

Education is considered to be one of the most important socio economic factors having an indirect influence on birth interval length through its impact on one or more of the bio-behavioral variables [11]. For example, in 38 of 51 countries with DHS data, women with no education were more likely than women with education to space births less than 3 years [16]. In this study, mothers with no education (25.5%) and primary education (54.8%) practice birth interval length less than 3 years when compared to those with secondary and above education (19.7%). Sometimes better educated women compress child bearing into fewer years to participate in non child bearing activities and hence have shorter birth intervals than less educated [11]. This might have brought the observed relation between birth interval and education among women with non education and primary education.

There are some urban-rural differentials with rural women less likely than urban women to have intervals over five years [11]. In 51 of 55 countries surveyed by the DHS, women who live in rural areas were more likely than women in urban areas to have birth intervals shorter than 3 years [16]. Similarly, in this study rural women were 2.66 times more likely to practice birth interval length less than 3 years as compared to their urban counterparts and have 4 months longer median birth interval length than rural women. Better social services and access to information, education and employment opportunities could have brought about variation by residence.

Birth interval showed difference by the age of the mother in which younger women had short birth interval more than older ones and this finding is similar with studies conducted in different places [11,16]. Similar to the pattern observed in Ethiopian demographic and health survey 2005 [1], the median number of months increase as the mothers age increases. On the other hand, as the mothers' age becomes older and older the proportion of mothers who practice short birth interval decreases and those who practice longer birth interval increases. This could be due to younger women being more likely to have children for a variety of reasons such as greater fecundity and being early on in the family building process. On the other hand, older women are later in their childbearing process and are likely to have achieved their desired family size and hence likely to have long subsequent spacing; they are also likely to be less fertile leading to longer spacing.

Different literatures show that, early marriage provides more years in which conception could occur in addition to its indirect effect through limited schooling and employment opportunities [7]. Unlike studies conducted in other places [7], women who had married at age 18 or more had one month shorter median birth interval length than those who had married at age less than 18 years. This discrepancy could be due to the difference in exposure to information on birth spacing practices. For example, exposure to information as well as knowledge on optimum birth spacing practices is relatively better among those who had married before age 18 than among those who had married at age 18 and above which might probably have led the observed difference in birth interval length practice between both groups.

Different studies have shown that, women are more likely to have their next child within 3 years if the previous child dies i.e. the longer the previous child survives, the less the effect on the subsequent birth interval [16]. In this study, women whose previous sibling has died had twelve months shorter birth interval. The Ethiopian demographic and health survey 2005 has shown similar effect of the survival nature of the index child on median birth interval length [1]. This could happen due to the desire of parents to replace a dead child sooner than if the child survives particularly when a child dies within the first year of life.

In the study conducted in Mozambique, the sex of the previous child does not seem to influence the length of the interval [11]. But couples who prefer son tend to have their next child soon after the birth of a daughter. Among 55 countries with demographic and health survey 2002, women were more likely to have a next child within 3 years after the birth of a daughter than after a son's birth [16]. In this study short intervals of less than 3 years follow in majority of the cases when the sex of the preceding child is female. Unlike the demographic and health survey 2002 and the current study, different findings were observed in a case of the study conducted in Mozambique. These variations could be due to the differences in sex preferences among the different cultures.

According to the Ethiopian demographic and health survey 2005, the median number of months increased when a wealth quartile is shifted from the lowest to the highest [1]. Similarly in this study, the median length of the birth interval grew as one shift from lowest quartile to highest quartile of the wealth index. Women in the better wealth category could probably have better access to information and education and hence could have longer birth intervals. But the multivariate analysis showed the opposite which may be attributed to recall bias to the actual birth interval and the validity and reliability of the wealth index measurement. Another reason might be women with low wealth index might be busy on their job to fulfill other basic needs resulting in delay of the birth interval. Such measurement bias may also have resulted inconsistent finding on the statistical association between birth interval and duration of breast feeding.

Actual birth intervals of women in most developing countries are shorter than the intervals they would prefer. Many women not only are unable to achieve their own reproductive goals but also are falling far short of the 3 to 5 years intervals that new evidence suggests are healthiest. In many sub-Saharan African countries, women are the furthest from achieving their preferred birth interval [16]. In the demographic and health survey analytical study conducted in 20 sub-Saharan countries, the median length of actual birth intervals was 33.7 months on average compared with preferred birth interval of almost 39.9 months [17]. Women in this study practiced birth intervals in average 7 months shorter than they would prefer. This wide gap showed a transition from high to low fertility; reproductive goals are changing but contraceptive behavior has yet to follow. One of the limitations of this study is there could be recall biases on reporting of actual birth intervals and breast feeding duration.

## Conclusion

Majority of the study subjects were aware of the optimal length of birth interval between two successive births. Accordingly, 60% of the respondents were knowledgeable about optimum birth interval. More than half (57%) of the women in reproductive age group have been practicing short birth interval below the recommended duration of optimal birth spacing. Regarding the median length of months between two successive births, women on average are 3 months behind what has been recommended as the healthiest birth interval.

Women who were rural resident, being student and daily worker in husbands' occupation, breast feeding for 7 to 12, 13 to 23 and 24 and above months, non use of modern contraceptives and highest wealth quartile were found to be significant predictors of short birth interval length. Women in the study area have been practicing on average 7 months shorter birth interval length than they would prefer otherwise and the preferred length of birth interval among the study subjects is significantly greater than actual birth interval length. Interventions to enhance contraceptive utilization behaviors among women in Lemo district would be helpful to narrow the gap between optimal and actual birth spacing.

## Competing interests

The authors declare that they have no competing interests.

## Authors' contributions

SY, MW and MA designed the study, analyzed the data and drafted the manuscript. EG was involved in the analysis of the data and critically reviewed the article.

All authors read and approved the final manuscript.

## Pre-publication history

The pre-publication history for this paper can be accessed here:

http://www.biomedcentral.com/1471-2393/11/38/prepub

## Supplementary Material

Additional file 1 **Questionnaire**. A questionnaire for assessing duration and determinants of birth interval among women of child bearing age Click here for file

## References

[B1] Central Statistical Agency [Ethiopia] and ORC Macro Ethiopia Demographic and Health Survey 2005 2006 Addis Ababa, Ethiopia and Calverton, Maryland, USA: Central Statistical Agency and ORC Macro

[B2] Herndn D Reynaldo M Robert K Nutrition, lactation, and birth interval components in rural Guatemala Am J Clin Nuir 1982 35 1468 1476 10.1093/ajcn/35.6.1468 7081128

[B3] Ethiopia CSA Summary and statistical report of the 2007 population and housing census 2008 Federal democratic republic of Ethiopia population census commission, Addis Ababa, Ethiopia 1 10

[B4] Samson G Mulugeta B Level and differentials of fertility in Awassa town, Southern Ethiopia Afr J Reprod Health 2009 13 1 93 112 20687268

[B5] Getu D Alemayehu W Estimation of the total fertility rates and proximate determinants of fertility in North and South Gondar zones, Northwest Ethiopia: An application of the Bongaarts' model Ethiop J Health Dev 2009 23 1 19 27

[B6] Yohannes D Factors influencing women's intention to limit child bearing in Oromia, Ethiopia Ethiop J Health Dev 2008 22 3 28 33

[B7] Ayanaw A Proximate determinants of birth interval length in Amhara region: the case of Fagita Lekoma district, Awi- zone 2008 Addis Ababa, Ethiopia

[B8] Yohannis F Yemane B Alemayehu W Differentials of fertility in rural Butajira Ethiop J Health Dev 2003 17 1 17 25

[B9] De Bruijn BJ Fertility: Theories, Models, Concepts 2006

[B10] Catalyst Consortium/Tahseen project Optimal birth spacing: an in-depth study of knowledge, attitudes and practices. Dec 2004

[B11] Saumya R John T Ian A Correlates of Inter-birth Intervals: Implications of Optimal Birth Spacing Strategies in Mozambique 2006 Population Council

[B12] Orji E Shittu A Makinde O Sule S Effect of prolonged birth spacing on maternal and perinatal outcome East Afr Med J 2004 81 8 388 391 15622931 10.4314/eamj.v81i8.9198

[B13] Nahla A Sarah L Amal Z Helping Egyptian women achieve optimal birth spacing intervals through fostering linkages between family planning and maternal and child health services. Sep 2008

[B14] Norton M New evidence on birth spacing: promising findings for improving newborn, infant, child, and maternal health Int J Gyn Obs 2005 89 51 56 10.1016/j.ijgo.2005.01.024 15820364

[B15] Abdolrahman R Majid M The determinants of birth interval in Ahvaz-Iran: A graphical chain modeling approach J Dat Sc 2007 5 555 57

[B16] Vidya S Ushma U Birth spacing: three to five saves lives. Population reports, series L, No. 13 2002 Baltimore, Johns Hopkins Bloomberg School of public health, population information program, summer 12469475

[B17] Rafalimanana H Charles W Gap between Preferred and Actual Birth Intervals in Sub-Saharan Africa: Implications for Fertility and Child Health. DHS Analytical Studies No. 2 2001 Calverton, Maryland: ORC Macro

