# Supplementary material for: Duration and determinants of birth interval among women of child bearing age in Southern Ethiopia
Source: BMC Pregnancy Childbirth. 2011 May 20;11:38. doi: 10.1186/1471-2393-11-38 (PMC3112064; doi:10.1186/1471-2393-11-38)
Supplement: Additional file 1 — Questionnaire. A questionnaire for assessing duration and determinants of birth interval among women of child bearing age [file 1471-2393-11-38-S1.DOC]

**ANNEXES**

**Questionnaire**

***Informed Consent***

Dear Respondent:

My name is ____________. This questionnaire is prepared to conduct a study on the socio-demographic, socio-economic and biological factors determining inter birth interval among WCBA in this area. You are selected and included in the study as part of the sample population to complete the questionnaire designed by the researcher. The information obtained in this study will be used only for research purposes. The data you will provide is very helpful to achieve the intended objectives of the study. Any information obtained will be kept strictly confidential and will not be exposed to any other body. But it may remind you some condition which you may not want to remember at all like death of your family member etc. Involvement in this study is optional and in voluntary basis and you can drop any individual question or the whole questionnaire. But your participation and contribution in the study is very important to come up with important findings which may help local health planners to intervene the problem locally.

Do you have any opinion regarding this study?

Do you agree to participate in this study?

Yes, continue No, thank you!

Name of the data collector________________________________ Sign________ Date______

Part I Socio-demographic Characteristics of the respondents

| **S.N** | **Questions** | **Response and Coding** | **Skip** |
| --- | --- | --- | --- |
| **101** | House code | ________________ |  |
| **102** | Residence | 1. Urban 2. Rural |  |
| **103** | Age of the mother in years | ________________ |  |
| **104** | Marital status | 1. Married 2. Single 3. Divorced 4. Widowed |  |
| **105** | At what age did you marry? | ________________ |  |
| **106** | Religion | 1. Orthodox 2. Protestant 3. Muslim 4. Catholic 5. Others (specify)____________ |  |
| **107** | Ethnicity | 1. Hadiya 2. Kembata 3. Guragie 4. Siltie 5. Amhara 6. Others (specify)____________ |  |
| **108** | Education of the mother | 1. Illiterate 2. Able to read and write 3. Elementary (1 - 6) 4. Junior (7 - 8) 5. Secondary (9 - 12) 6. Tertiary (12+) |  |
| **109** | Education of the husband | 1. Illiterate 2. Able to read and write 3. Elementary (1 - 6) 4. Junior (7 - 8) 5. Secondary (9 - 12) 6. Tertiary (12+) | Skip to |
| **110** | Occupation of the mother | 1. Employee (GO/NGO) 2. House wife 3. Merchant 4. Student 5. Farmer 6. Daily worker 7. Others (Specify)_________ |  |
| **111** | Occupation of the husband | 1. Employee (GO/NGO) 2. Merchant 3. Student 4. Farmer 5. Daily worker 6. Others (Specify)___ | Skip to |

Part II Knowledge on Birth Interval

| **S.N** | **Questions** | **Response and Coding** | **Skip** |
| --- | --- | --- | --- |
| **201** | Have you heard about optimal birth interval between two consecutive births? | 1. Yes  2. No | If no go to Q**203** |
| **202** | If yes to question no 201, what is the optimum number of months or years between two successive births? | 1. Below three years 2. Three to five years 3. Above five years 4. I don’t know |  |
| **203** | Does adequate/optimum birth spacing have a **healt**h advantages? | 1. Yes 2. No 3. Don’t Know | If no or DK go to Q**205** |
| **204** | If yes to question no 203, whom do you think have a health advantages? |  |  |
|  | 204.1. The mother? | 1. Yes 2. No 3. Don’t Know |  |
|  | 204.2. The child? | 1. Yes 2. No 3. Don’t Know |  |
|  | 204.3. Both? | 1. Yes 2. No 3. Don’t Know |  |
| **205** | Does short birth interval have a health disadvantages? | 1. Yes 2. No 3. Don’t Know | If no or DK go to Q**301** |
| **206** | If yes to question no 205, whom do you think have a health disadvantages? |  |  |
|  | 206.1. The mother? | 1. Yes 2. No 3. Don’t Know |  |
|  | 206. 2. The child? | 1. Yes 2. No 3. Don’t Know |  |
|  | 206. 3. Both? | 1. Yes 2. No 3. Don’t Know |  |

Part III Birth History of the respondents

| **S.N** | **Questions** | | | | **Response and Coding** | | | **Skip** | |
| --- | --- | --- | --- | --- | --- | --- | --- | --- | --- |
| **301** | How many children have you ever born alive? | | | | Males ____ Females_____ | | |  | |
| **302** | Have you ever given birth to any child who died later? | | | | 1. Yes 2. No | | | If no go to Q**304** | |
| **303** | If yes, how many of your children died? | | | | Males _____ Females_____ | | |  | |
| **304** | At the time you became pregnant with the last child did you want to have more child? | | | | 1. Yes 2. No | | | If no go to Q**308** | |
| **305** | If yes to question no 304, was your preference to become pregnant then or wait until later? | | | | 1. to become pregnant then 2. to wait until later | | | If “1” go to Q**308** | |
| **306** | If your preference is to wait until later, how long did you prefer to wait? | | | | _____________ | | |  | |
| **307** | What is the reason to become pregnant then while preferring to wait until later? | | | | _____________ | | |  | |
| **308**. Birth Order | | **1.**  Sex  1. Male  2. Female | **2**. In what month and year did (name) born? | **3**. Is he /she alive?  1. Yes  2. No | | **4**. If died, how old was (name) he/she died  1----------year  2---------Month | **5**. Current age | | **309**. Birth interval in months |
| **308.1**.Last child | |  |  |  | |  |  | |  |
| **308.2**. previous to last child | |  |  |  | |  |  | |

Part IV Breast feeding practice

| **S.N** | **Questions** | **Next to Last Child (Response)** | **Skip** |
| --- | --- | --- | --- |
| **401.** | Did you breast fed previous to last child (name)? | 1. Yes  2. Never breast fed | If “never” go to Q**404** |
| **402.** | If yes to question 401, for how long was (name) breastfed? | ________months |  |
| **403.** | Reason for stopping breast feeding? | 1. The child being old enough  2. New pregnancy  3. The mother was sick  4. Other(specify)_____ |  |
| **404.** | When do you think breast feeding should stop completely? | After _______ months. |  |

Part V. Knowledge and practice of modern contraceptive use

| **S.N** | **Questions** | **Response and Coding** | **Skip** |
| --- | --- | --- | --- |
| **501** | Do you know any modern method that women and men can use to delay or avoid pregnancy? | 1. Yes 2. No | If no go to Q**601** |
| **502** | If yes for q501, which of the following methods do you know about? |  |  |
|  | **502.1**. Pills | 1. Yes 2. No |  |
|  | **502. 2**.Injectable | 1. Yes 2. No |  |
|  | **502. 3**. Condom | 1. Yes 2. No |  |
|  | **502. 4**. Implants | 1. Yes 2. No |  |
|  | **502. 5**. IUD | 1. Yes 2. No |  |

| **503** | Have you been using any of the modern methods before the conception of your last child? | 1. Yes 2. No | If no go to Q**506** |
| --- | --- | --- | --- |
| **504** | If yes to question no **503**, what was the purpose? | 1. Birth spacing  2. Limiting birth |  |
| **505** | If yes to question no **503,** which of the following modern methods did you use? | 1. Pills 2. Injectables 3. Condom 4. Implanta 5. IUCD |  |
| **506** | Are you using any of the modern methods now? | 1. Yes 2. No | If no go to Q**508** |
| **507** | If yes to question **503** or **506**, from where have you got family planning service? | 1. Health post 2. Health center  3. Hospital 4. Private sector |  |
| **508** | If you were not using any contraceptive method to delay or avoid pregnancy, would you tell me the main reason? | 1. Desire for more children 2. Health problem 3. Religious reason 4. Moral and cultural reason 5. Lack of information about   contraception   1. FP service not available 2. Others /specify/__________ |  |

Part VI: Socio-economic characteristics of respondents

| **S.N** | **Questions** | **Response and Coding** | **Skip** |
| --- | --- | --- | --- |
| **601** | What is the main source of drinking water for members of your household?  *Circle ONLY ONE answer* | 1. piped water 2. protected Dug well 3. unprotected dug well 4. protected spring 5. unprotected spring 6. rainwater 7. tanker truck 8. surface water (River, pond) 9. bottled water 10. others__________________ |  |
| **602** | Do you treat your water in any way to make it safer to drink? | 1. Yes 2. No | If no go to Q**604** |
| **603** | What do you usually do to the water to make it safer to drink?  *Circle ALL applicable answers* | 1. boil 2. add bleach/chlorine 3. strain through a cloth 4. use water filter (ceramic/Sand/ compo 5. let it stand and settle 6. others _______________ |  |
| **604** | What kind of toilet facility do members of your household usually use?  *Circle ONLY ONE answer* | 1. flush or pour flush toilet 2. ventilated improved pit latrine (VIP) 3. pit latrine with slab 4. pit latrine without slab/ open pit 5. No facility/bush/field 6. others (specify _____________ |  |
| **605** | Does your household have: | 1. Electricity? 2. A watch? 3. A radio? 4. A television? 5. A mobile telephone 6. A non-mobile telephone? 7. A refrigerator? 8. A table? 9. A chair? 10. A bed? 11. An electric mitad? |  |
| **606** | What type of fuel does your household mainly use for cooking?  *Circle ONLY ONE answer* | 1. electricity 2. biogas 3. kerosene 4. charcoal 5. wood 6. straw/shrubs/grass 7. animal dung 8. others (specify)____ |  |

| **S.N** | **Questions** | **Response and Coding** | **Skip** |
| --- | --- | --- | --- |
| **607** | Do you have separate room which is used as kitchen? | 1. Yes 2. No |  |
| **608** | Main material of the floor (observation)  *Circle ONLY ONE answer* | 1. earth/ mud 2. wooden 3. ceramic tiles 4. cement/bricks 5. other [specify]_____________ |  |
| **609** | Main material of the roof (observation)  *Circle ONLY ONE answer* | 1. thatch/leaf 2. plastic sheets 3. wood 4. corrugated iron sheet 5. cement 6. other [specify]______________ |  |
| **610** | Main material of the walls (observation)  *Circle ONLY ONE answer* | 1. wooden and mud 2. wood/sticks 3. cement 4. stone with lime/cement 5. bricks 6. WOOD plank/SHINGLES 7. other [specify]____________________ |  |
| **611** | What is now the primary source of income for this household?  *Circle ONLY ONE answer* | 1. farming, including cash crops  2. livestock  3. employment/salary  4. petty trading (including sale of fire-  wood, charcoal, grass, local brewery)  5. daily labor  6. handicrafts/artisan  7. remittances |  |
| **612** | Does this household own any land that can be used for agriculture? | 1. Yes 2. No | If no go to Q**614** |
| **613** | If yes how much land does your household own? | ___________Timad |  |
| **614** | Does this household own any livestock, herds, or farm animals? | 1. Yes 2. No | If no stop |
| **615** | How many of the following animals do this household own?  **[PROBE AND MARK THAT ALL APPLY, MULTIPLE ANSWER IS POSSIBLE]** | 1. cows_________ 2. oxen, or bulls____________ 3. calves ____________ 4. horses/donkeys/ mules_________ 5. sheep and Goats____________ 6. chickens_____________ |  |

[Ayanaw A. Proximate determinants of birth interval length in Amhara region: the case of Fagita Lekoma district, Awi- zone, Addis Ababa, Ethiopia, 2008]
